# Supplementary material for: MetaQTL: a package of new computational methods for the meta-analysis of QTL mapping experiments
Source: BMC Bioinformatics. 2007 Feb 8;8:49. doi: 10.1186/1471-2105-8-49 (PMC1808479; doi:10.1186/1471-2105-8-49)
Supplement: Additional File 7 — MetaQTL Package : jar file and tutorial. This Zip archive contains both the MetaQTL JAR file and the files of the tutorial. [file 1471-2105-8-49-S7.zip › org.inra.metaqtl/doc/org/metaqtl/IBioLGroupProperties.html]

IBioLGroupProperties


|  |  |  |
| --- | --- | --- |
| **Overview** \| **Package** \| **Class** \| **Use** \| **Tree** \| **Deprecated** \| **Index** \| **Help** | |  |
| **PREV CLASS**   **NEXT CLASS** | **FRAMES**    **NO FRAMES**     **All Classes** |  |
| SUMMARY: NESTED \| FIELD \| CONSTR \| METHOD | DETAIL: FIELD \| CONSTR \| METHOD |  |


---


## org.metaqtl Interface IBioLGroupProperties

---

``` public interface IBioLGroupProperties ```

This interface defines the key of properties which can
be handled by a org.thalia.bio.entity.IBioLGroup.

---

| **Field Summary** | |
| --- | --- |
| `static java.lang.String` | `CHISQUARE`             The key for the chisquare value. |
| `static java.lang.String` | `DOF`             The key for the number of degrees of freedom. |
| `static java.lang.String` | `PVALUE`             The key for the p-value. |

**Field Detail**

### CHISQUARE

```
static final java.lang.String CHISQUARE
```

:   The key for the chisquare value.

    **See Also:**: Constant Field Values

---


### PVALUE

```
static final java.lang.String PVALUE
```

:   The key for the p-value.

    **See Also:**: Constant Field Values

---


### DOF

```
static final java.lang.String DOF
```

:   The key for the number of degrees of freedom.

    **See Also:**: Constant Field Values


---


|  |  |  |
| --- | --- | --- |
| **Overview** \| **Package** \| **Class** \| **Use** \| **Tree** \| **Deprecated** \| **Index** \| **Help** | |  |
| **PREV CLASS**   **NEXT CLASS** | **FRAMES**    **NO FRAMES**     **All Classes** |  |
| SUMMARY: NESTED \| FIELD \| CONSTR \| METHOD | DETAIL: FIELD \| CONSTR \| METHOD |  |


---
